# *CircRNA_0075723* protects against pneumonia-induced sepsis through inhibiting macrophage pyroptosis by sponging miR-155-5p and regulating SHIP1 expression

**DOI:** 10.3389/fimmu.2023.1095457

**Published:** 2023-02-27

**Authors:** Dianyin Yang, Dongyang Zhao, Jinlu Ji, Chunxue Wang, Na Liu, Xiaowei Bao, Xiandong Liu, Sen Jiang, Qianqian Zhang, Lunxian Tang

**Affiliations:** ^1^ Department of Internal Emergency Medicine, Shanghai East Hospital, Tongji University School of Medicine, Shanghai, China; ^2^ Medical School, Tongji University, Shanghai, China; ^3^ Department of Nephrology, Shanghai East Hospital, Tongji University School of Medicine, Shanghai, China

**Keywords:** CircRNA_0075723, miR-155-5p, SHIP1, pyroptosis, pneumonia-induced sepsis, THP-1

## Abstract

**Introduction:**

Circular RNAs (circRNAs) have been linked to regulate macrophage polarization and subsequent inflammation in sepsis. However, the underlying mechanism and the function of circRNAs in macrophage pyroptosis in pneumonia-induced sepsis are still unknown.

**Methods:**

In this study, we screened the differentially expressed circRNAs among the healthy individuals, pneumonia patients without sepsis and pneumonia-induced sepsis patients in the plasma by RNA sequencing (RNA-seq). Then we evaluated macrophage pyroptosis in sepsis patients and in vitro LPS/nigericin activated THP-1 cells. The lentiviral recombinant vector for circ_0075723 overexpression (OE-circ_0075723) and circ_0075723 silence (sh-circ_0075723) were constructed and transfected into THP-1 cells to explore the potential mechanism of circ_0075723 involved in LPS/nigericin induced macrophage pyroptosis.

**Results:**

We found circ_0075723, a novel circRNA that was significantly downregulated in pneumonia-induced sepsis patients compared to pneumonia patients without sepsis and healthy individuals. Meanwhile, pneumonia-induced sepsis patients exhibited activation of NLRP3 inflammasome and production of the pyroptosis-associated pro-inflammatory cytokines IL-1β and IL-18. circ_0075723 inhibited macrophage pyroptosis via sponging miR-155-5p which promoted SHIP1 expression directly. Besides, we found that circ_0075723 in macrophages promoted VE-cadherin expression in endothelial cells through inhibiting the release of NLRP3 inflammasome-related cytokines, IL-1β and IL-18, and protects endothelial cell integrity.

**Discussion:**

Our findings propose a unique approach wherein circ_0075723 suppresses macrophage pyroptosis and inflammation in pneumonia-induced sepsis via sponging with miR-155-5p and promoting SHIP1 expression. These findings indicate that circRNAs could be used as possible potential diagnostic and therapeutic targets for pneumonia-induced sepsis.

## Introduction

Sepsis is defined as a life-threatening organ dysfunction caused by a dysregulated host response to infection (1). Despite significant advances of sepsis therapy in the past decade, sepsis remains the primary reason for death in the intensive care unit (ICU) (2). Infection in the respiratory system is the most common of sepsis, which accounts for about 50%. Meanwhile pulmonary infections lead to nearly 30% mortality of patients with sepsis, much higher than infections from other sources (3, 4). However, the mechanisms driving pneumonia-induced sepsis remain poorly understood. Macrophage death is critical to the pathophysiology of pneumonia and related sepsis (5, 6). Of note, pyroptosis, a sort of programmed cell death driven by NLRP3 inflammasome activation, is a major contributor to sepsis. It is characterized by formation of cell membrane pores and the production of inflammatory factors IL-1β and IL-18 (7). In reaction to pathogen-associated molecular patterns (PAMPs) and damage-associated molecular patterns (DAMPs), NLRP3 is activated and oligomerized through NACHT domain, which then recruits apoptosis-associated speck-like protein containing a CARD (ASC) and pro-caspase 1 to form NLRP3 inflammasome. This results in the transformation of pro-caspase1 into active caspase1. The active caspase1 then converts pro-IL-1β and pro-IL-18 to their active forms. With its pore-forming activity, Caspase1 also cleaves gasdermin D (GSDMD) into N-terminal form (N-GSDMD). Inflammatory factors (IL-1β and IL-18) are ultimately released from pores formed by N-GSDMD. Increasing evidence have indicated that NLRP3 inflammasome and pyroptosis in macrophages are essential for the occurrence and development of sepsis (8, 9). However, whether macrophage pyroptosis is involved in pneumonia-induced sepsis and the precise regulatory mechanisms of macrophage pyroptosis remain not clear.

Circular RNAs (circRNAs) are covalently closed single-strand RNAs generated by mRNA back-splicing, which comprise a widespread subtype of non-coding RNAs (10). The main function of circRNAs is regulation of transcription and translation of mRNA by sponging miRNAs (11). Till now, circRNAs have been implicated in numerous areas of biological processes, including cell differentiation, apoptosis, autophagy, and proliferation, which are all closely related to septic pathogenesis (12). We previously reported global changes of circRNAs and the circRNA-miRNA-mRNA networks in pulmonary macrophages activation from cecal ligation and puncture (CLP)-induced acute respiratory distress syndrome (ARDS) mice model by microarray analysis, suggesting that circRNAs are required for macrophages function and the development of ARDS (13). Further, we have revealed that *circN4bp1* facilitated sepsis-induced ARDS through promoting macrophage polarization by means of miR-138-5p/EZH2 axis *in vivo* and ex vivo (14). Recent reports have implicated circular RNAs in the regulation of macrophage pyroptosis. *CircACTR2* is identified to promote macrophage pyroptosis and the subsequent fibrosis (15). Inhibition of *circ_0029589* by IFN regulatory Factor-1 (IRF-1) may also promote macrophage pyroptosis and inflammation in patients with acute coronary syndrome (ACS) (16). However, it remains unknown whether circRNAs regulate macrophage pyroptosis in sepsis, especially pneumonia-induced sepsis. In this investigation, we screened for differential expression circRNAs in plasma of healthy individuals, pneumonia patients without sepsis, and pneumonia-induced sepsis patients using RNA-seq and recognized the significantly downregulated circRNA, *circ_0075723*, which is generated from the exons of gene *NUP153*. We also showed that *circ_0075723* acted as a negative regulator of macrophage pyroptosis and inflammatory damage in pneumonia-induced sepsis, in addition to the pathways associated with miR-155-5p and SHIP1. Our findings present new insights of circRNAs into the regulation of macrophage pyroptosis and provide possible treatment targets for pneumonia-induced sepsis.

## Materials and methods

### Clinical samples collection

This study was authorized by the Research Ethics Board of East Hospital, Tongji University (Shanghai, China). All recruited patients or their authorized family members were provided with a consent form. Peripheral blood (4ml) was taken from 7 eligible patients with pneumonia-induced sepsis, 7 pneumonia patients without sepsis and 7 healthy donors. The participants’ clinical parameters are shown in **Supplementary Table 1**. The pneumonia patients were classified as sepsis according to the Surviving Sepsis Campaign definitions (17) from the emergency and/or general intensive care unit (ICU) of East Hospital. The pneumonia patients without sepsis who came from emergency internal medicine ward of East Hospital and healthy volunteers came to East hospital for routine physical examination. Pneumonia was defined by a new pulmonary infiltrate on chest radiograph accompanied with at least one of the following signs (18): (a) the presence of cough, sputum production, and dyspnea; (b) core body temperature > 38.0°C; (c) peripheral white blood cell counts > 10 × 10^9^/L or < 4 × 10^9^/L. Among the 21 samples, 3 sepsis samples, 3 pneumonia samples and 3 healthy people samples were used for RNA sequencing analysis, and the remaining samples were used for subsequent tests.

### Cell extraction

Peripheral blood mononuclear cells (PBMCs) were isolated from peripheral blood according to the protocol as previously reported (19) and CD14^+^ monocytes were sorted from PBMCs with a magnetic cell sorting system (Miltenyi Biotec, Germany). PBMCs were added with CD14 Microbeads(20µl/10^7^cells), and then the CD14^+^ monocytes were magnetically labeled with CD14 Microbeads. When PBMCs passed through a MACS column, the magnetically labeled CD14^+^ monocytes were retained within the column, and then CD14+monocytes were extracted as positively selected cell fraction.

### RNA sequencing analysis

Plasma from patients with pneumonia-induced sepsis, pneumonia patients without sepsis, and healthy individuals was isolated using TRIzol reagent (Invitrogen, USA) per the manufacturer’s instructions. NanoDrop ND-1000 was utilized to measure the RNA’s purity and concentration (NanoDrop Thermo). Through denaturing agarose gel electrophoresis, the RNA integrity of the samples was evaluated. The rRNA was removed using the Ribo-Zero rRNA Removal Kit (Illumina, San Diego, CA, USA). Cloud-Seq Biotech (Shanghai, China) performed the high-throughput whole transcriptome sequencing and subsequent bioinformatics analysis as previously reported (20). The sequencer Illumina HiSeq 6000 was used to obtain paired-end readings. The circular RNA was detected and identified using DCC software (v0.4.4) and the identified circular RNA was annotated using the circBase database and Circ2Tuits. Edger software (v3.16.5) was utilized to identify circRNAs with differential expression.

### RNA extraction and quantitative Real-Time PCR (qRT-PCR)

Total RNA (2µg) was extracted using TRIzol (Invitrogen, USA) followed by reverse transcription of mRNAs and circRNAs using PrimeScript II 1st Strand cDNA Synthesis Kit (Takara, Japan) per the standard manufacturer’s instructions. qRT-PCR assay was performed to measure mRNAs and circRNAs expression with SYBR^®^ Premix Ex Taq™ II (Takara, Japan) using the Roche 480 Real Time PCR System. GAPDH (encoding glyceraldehyde-3-phosphate dehydrogenase) was used as an internal control for circRNAs and mRNAs, and U6 was employed as an endogenous control for the miRNAs. Relative quantification (2^−ΔΔCT^) was used for result analysis. All the primers used were included in **Supplementary Table 2**.

### Cell culture and transfection

The human monocytic leukemia cell line THP-1 was purchased from Chinese Academy of Sciences (Shanghai, China) and was grown in RPMI-1640 medium supplemented with 10% fetal bovine serum and 1% penicillin and streptomycin. THP-1 cells were differentiated into macrophages for 3 hr in the presence of 100 nM phorbol myristate (PMA) and replated. For stimulation, cells were primed with different or indicated concentrations (0.1, 0.5 and 1 μg/ml) LPS for 4 hrs in Opti-MEM, then stimulated with 10 μM nigericin for 2 hrs. GenePharma designed and produced the *Circ_0075723* overexpression vector, miR-155-5p mimic, SHIP1 overexpression vector and *circ_0075723* silence vector (sh-*circ_0075723*) (Shanghai, China). THP-1 cells were transfected with overexpression vector (2 μg), miRNA mimic (25 nM) or shRNA (25 nM) using Lipofectamine 3000 (Invitrogen) per the manufacturer’s instructions 24h before LPS/nigericin stimulation. After different stimulations, the supernatants of THP-1 cells were collected for IL-1β and IL-18 analyses or applied to culture human lung microvascular endothelial cells (HLMVEC) (Chinese Academy of Sciences, China) for 24 hours.

### Fluorescence *in situ* hybridization (FISH) assay

The location of *circ_0075723* in THP-1 cells is determined by FISH. THP-1 cells are fixed with 4% paraformaldehyde and gradient dehydrated with ethanol. Fluorescent-labeled probe (1 µM) for *circ_0075723* is applied during hybridization. We use DAPI (Beyotime, Shanghai, China) to stain the nucleus of macrophages.

### Luciferase reporter assay

The wild-type (WT) sequence and mutant-type (MUT) sequences (binding site mutation with miR-155-5p) of *circ_0075723* and SHIP1 were amplified and cloned into PmirGLO reporter plasmid, respectively. The fusion plasmid was cotransfected with either miR-155-5p or miR-NC into HEK293T cells. 48 hours after transfection, the luciferase activity was measured using Picagene Dual SeaPansy luminescence kit (Toyo Inc., Japan) according to the manufacturer’s instructions as reported (21).

### RNase R digestion RNA stability

4 μg total RNA from THP-1 cells was either untreated (control) or treated with 20 units of RNase R (Epicenter; USA, RNR07250) in the presence of 1× reaction buffer and incubated for 30 min at 37°C. RNA was extracted using acid phenol-chloroform after digestion (5: 1). Then, reverse transcription and qRT-PCR were performed, as described in the RNA extraction and qRT-PCR section. THP-1 cells (1 ×10^5^) were placed in 24-well plates and treated with 250 ng/ml actinomycin D (Act D, Sigma) added to the cell culture medium. The levels of *circ_0075723* and *NUP153* were measured at 0, 8, 12, and 24 hrs.

### RNA pull-down assay

Biotin-labeled *circ_0075723* probe and oligo probe were obtained from Ribobio. THP-1 cells transfected with *circ_0075723* probe or oligo probe were lysed and used for pull-down assay using the Pierce Magnetic RNA ProteinPull-down Kit (Thermo Fisher Scientific) in accordance with the instructions. qRT-PCR was used to detect the expression of specified miRNAs.

### ELISA analysis

ELISAs were performed to measure the concentrations of IL-18 and IL-1β protein from supernatants according to the manufacturer’s instructions (R&D Systems).

### Immunoblotting analysis

Immunoblotting analysis was performed as described previously (19, 22). Densitometry analysis of immunoblot results was conducted by using ImageJ software. The results of three replicated experiments are expressed as mean ± standard deviation (SD) (primary antibodies are listed in **Supplementary Table 3**).

### Statistical analysis

All experiments were done in triplicates and replicated at least three times and all experimental data are presented as the means ± SD. The two-tailed Student t-tests were used for comparisons between two groups, and one-way or two-way analysis of variance (ANOVA) were used for multifactorial comparisons. Statistical analyses were performed with SPSS 20.0 software (SPSS Inc., Chicago, IL, USA) or GraphPad Prism 8.0 (GraphPad Software, La Jolla, CA, United States). A value of P < 0.05 was considered to indicate a statistically significant difference.

## Results

### Specifical expression profiles of circRNAs in pneumonia-induced sepsis

**Figure 1:**
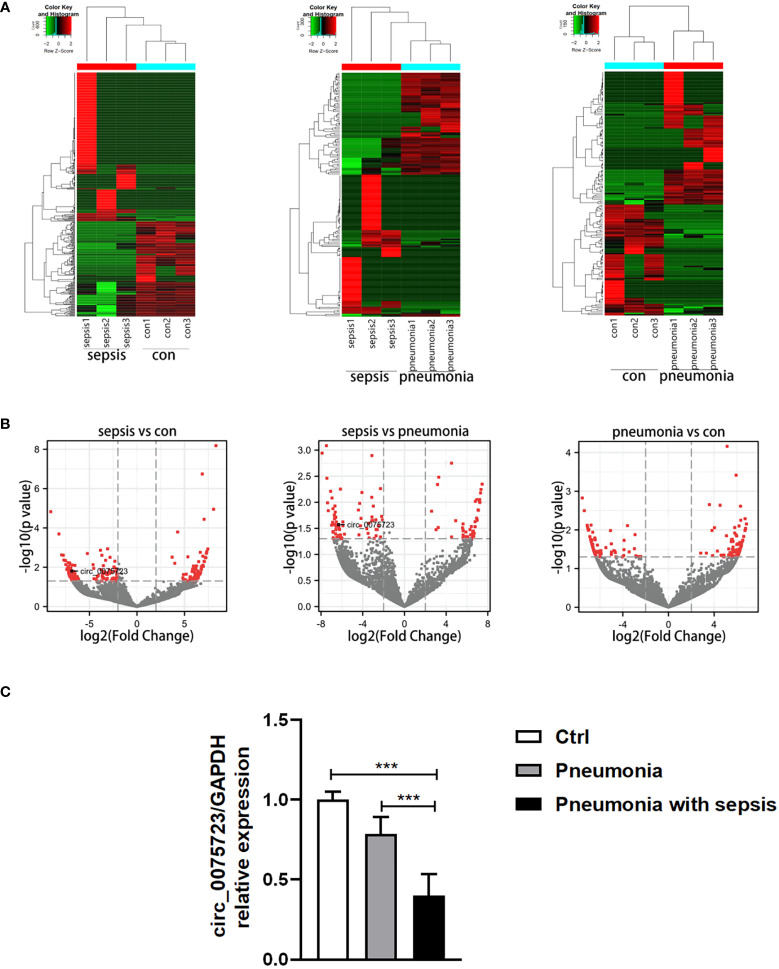
Specifical expression profiles of circRNAs in pneumonia-induced sepsis. **(A)** Hierarchical cluster analysis of differentially expressed circRNAs between the two compared groups of plasma. **(B)** Volcano plots showing the differentially expressed circRNAs among the three groups [Plot of circRNA expression log2‐transformed fold‐changes (x‐axis) vs ‐log10 P‐value (y‐axis)]. The red dots represent the circRNAs having fold change > 2.0 and P < 0.05 between the two compared groups of plasma. **(C)** qRT-PCR analysis of circ_0075723 expression in CD14+ monocytes among the pneumonia-induced sepsis, pneumonia without sepsis and healthy control group. Each group has 4 samples. Data are presented as means ± SD; significant difference was identified with one-way ANOVA. ***p < 0.001 vs. Control or Pneumonia.

To determine circRNAs expression profiles and to identify those that are differentially expressed in pneumonia-induced sepsis, we selected healthy people and pneumonia patients without sepsis as controls and performed RNA-seq analysis of circRNA in the plasma of these three groups. In total, 32,229 circRNAs were expressed in the plasma samples among the healthy people, pneumonia patients without sepsis and pneumonia-induced sepsis patients (**Supplementary Table 4**). Using the cutoff values of fold change > 2.0 and P < 0.05, 382 circRNAs showed significantly differential expression between the pneumonia-induced sepsis patients and healthy people, including 233 circRNAs that were upregulated and 149 circRNAs that were downregulated (**Supplementary Tables 5, 6**) (**Figures 1A, B**, **Supplementary Figure 1**). Meanwhile, 172 differentially expressed circRNAs were detected between the pneumonia-induced sepsis patients and pneumonia patients without sepsis, in which 98 of them were upregulated and 74 were downregulated (**Supplementary Tables 7, 8**) (**Figures 1A, B**). Gene ontology (GO) and Kyoto Encyclopedia of Genes and Genomes (KEGG) analysis of differentially expressed circRNAs were showed in **Supplementary Figure 2**. GO enrichment analysis showed that the differentially expressed circRNAs were involved in the biological processes, such as cell energy metabolism and histone modification. On the other hand, KEGG analysis revealed that RNA transport and MAPK signaling pathway, which is associated with inflammatory activation of macrophages, was related to the differential expression of circRNAs. Of all the differential circRNAs, we chose markedly downregulated *circ_0075723* for next investigations because one of its most likely targeted gene, SHIP1(Src homology 2 domain–containing inositol-5-phosphatase 1), had been previously documented to be one of the negative regulators of TLR4 signaling which was involved in regulating NLRP3 inflammasome activation and pyroptosis (23, 24). Furthermore, we validated the expression of *circ_0075723* in CD14^+^ monocytes by qRT-PCR among the three groups and found that *circ_0075723* was significantly downregulated in pneumonia-induced sepsis patients comparing to the other two groups (**Figure 1C**). Taken together, we screened multiple differently expressed circRNAs in pneumonia-induced sepsis compared to healthy people and pneumonia without sepsis by RNA-seq and validated the significant downregulation of *circ_0075723* in pneumonia-induced sepsis, the differential expression of circRNAs from sepsis suggest possible functions of circRNAs in pathogenesis of sepsis.

### The characterization of the *circ_0075723*

**Figure 2:**
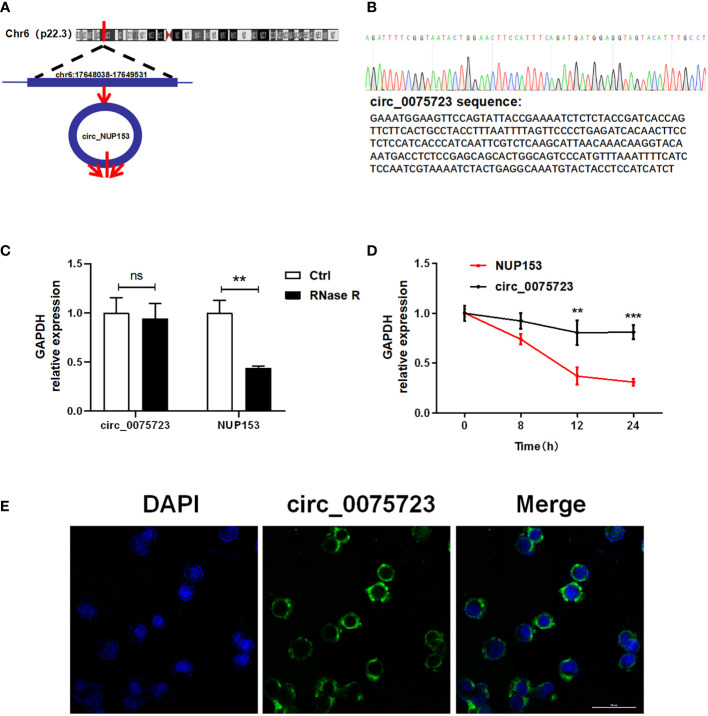
The characterization of the circ_0075723. **(A)** The location of circ_0075723 in genome. **(B)** Sanger sequencing showing the “head-to-tail” splicing of circ_0075723 in THP-1 cell. **(C)** qRT-PCR analysis of the expression of circ_0075723 and NUP153 in THP-1 cells after treatment with RNase R. Data are presented as means ± SD; significant difference was identified with two-way ANOVA. **p < 0.01; ns: no significant. **(D)** qRT-PCR analysis of the expression of circ_0075723 and NUP153 in THP-1 cells after treatment with actinomycin **(D)** Data are presented as means ± SD; significant difference was identified with Student *t*-tests. **p < 0.01, ***p < 0.001 **(E)** RNA FISH for circ_0075723. Nuclei were stained with DAPI.

*Circ_0075723*, located at chr6:17648038-17649531, which is derived from the human *NUP153* gene and generated by back-splicing mechanism (**Figure 2A**). The sequence was located at the back-splice junction location of *circ_0075723* according to Sanger sequencing (**Figure 2B**). Further, we treated THP-1 cells with RNase R exonuclease or actinomycin D to confirm *circ_0075723* authenticity and found that the expression of *circ_0075723* exhibited RNase R (**Figure 2C**) and actinomycin D resistance (**Figure 2D**), while that of *NUP153* mRNA was significantly decreased. This indicated *circ_0075723* was stable in THP-1 cells. We then investigated the sub-cellular location of *circ_0075723*. By RNA fluorescence *in situ* hybridization (FISH) assays, we found *circ_0075723* was mainly localized in the cytoplasm (**Figure 2E**). These studies indicated that *circ_0075723* as a circRNA, its biological stability may be advantageous to its function.

### Pyroptosis is activated following pneumonia-induced sepsis and *Circ_0075723* inhibits pyroptosis of THP-1 *in vitro*

**Figure 3:**
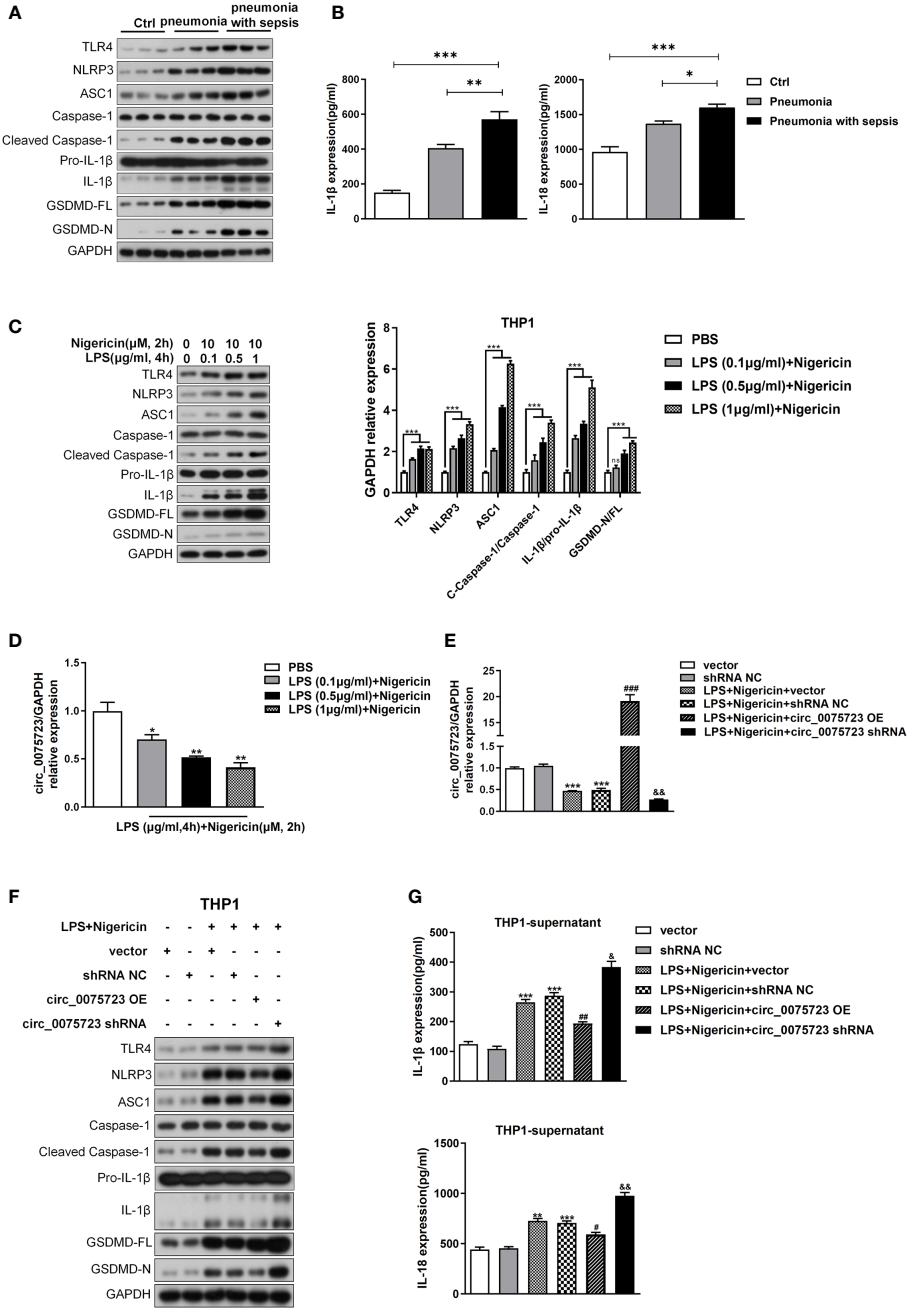
Pyroptosis is activated following pneumonia-induced sepsis and Circ_0075723 inhibits pyroptosis of THP-1 *in vitro*. **(A)** Western blot analysis of TLR4, NLRP3, ASC1, caspase1, cleaved caspase-1, Pro-IL1β, IL-1β, GSDMD and GAPDH in CD14+ monocytes from pneumonia-induced sepsis, pneumonia without sepsis and healthy people. **(B)** ELISA of IL-18 and IL-1β in the plasma from pneumonia-induced sepsis, pneumonia without sepsis and healthy people. Each group has 4 samples. Data are presented as means ± SD; significant difference was identified with one-way ANOVA. *p < 0.05 vs. Pneumonia; **p < 0.01 vs. Pneumonia; ***p < 0.001 vs. Control. **(C)** Western blot analysis of TLR4, NLRP3, ASC1, caspase1, cleaved caspase-1, Pro-IL1β, IL-1β, GSDMD and GAPDH in THP-1 cells primed with different doses of LPS (0.1, 0.5 and 1 μg/ml) for 4 h and stimulated with nigericin (10 μM) for 2h. Data are presented as means ± SD; significant difference was identified with two-way ANOVA. ***p < 0.001 vs. PBS; ns: no significant. **(D)** qRT-PCR analysis of the expression of circ_0075723 primed with different doses of LPS (0.1, 0.5 and 1 μg/ml) for 4 h and stimulated with nigericin (10 μM) for 2h in THP-1 cells. Data are presented as means ± SD; significant difference was identified with Student *t*-tests. *p < 0.05 vs. PBS; **p < 0.01 vs. PBS. THP-1 cells were transfected with vector or shRNA scrambled control (shRNA NC) or were transfected with circ_0075723-overexpressing lentivirus plasmids (OE-circ_0075723), sh-circ_0075723-expressing lentivirus plasmids (sh-circ_0075723), vector or shRNA scrambled control (shRNA NC) and then were primed with LPS (1 μg/ml) for 4 h and stimulated with nigericin (10 μM) for 2h. **(E)** qRT-PCR analysis of the expression of circ_0075723 in THP-1 cells. Data are presented as means ± SD; significant difference was identified with Student *t*-tests. ***p < 0.001 vs. vector or shRNA NC; ###p < 0.001 vs. LPS/nigericin + Vector; &&p < 0.01 vs. LPS/nigericin + shRNA NC. **(F)** Western blot analysis of TLR4, NLRP3, ASC1, caspase1, cleaved caspase-1, Pro-IL1β, IL-1β, GSDMD and GAPDH in THP-1 cells. **(G)** ELISA of IL-18 and IL-1β in THP-1 supernatant. Data are presented as means ± SD; significant difference was identified with Student *t*-tests. **p < 0.01 vs. vector; ***p < 0.001 vs. vector or shRNA NC; #p < 0.05 vs. LPS/nigericin + Vector; ##p < 0.01 vs. LPS/nigericin + Vector; &p < 0.05 vs. LPS/nigericin + shRNA NC; &&p < 0.01 vs. LPS/nigericin + shRNA NC.

We then analyzed *circ_0075723* function in pneumonia-induced sepsis. Given that pyroptosis, a typical inflammatory cell death, is a major features/characteristics of sepsis (7, 9), we wondered whether *circ_0075723* was involved in the regulation of macrophage pyroptosis. Firstly, we found that CD14+ monocytes from pneumonia-induced sepsis patients had considerably greater levels of the proteins TLR4, NLRP3, ASC1, cleaved caspase-1, IL-1, and GSDMD in contrast to pneumonia patients without sepsis and healthy people (**Figure 3A**, **Supplementary Figure 3A**). In addition, pneumonia-induced sepsis patients showed a markedly enhanced expression of IL-1β and IL-18 from plasma in comparison to pneumonia patients without sepsis and healthy people (**Figure 3B**). These results indicated that pyroptosis is activated in pneumonia-induced sepsis. Due to the markedly downregulated expression of *circ_0075723* in CD14^+^ monocytes from pneumonia-induced sepsis patients, we next examined the specific role of *circ_0075723* in pyroptosis of pneumonia-induced sepsis. By usage of different doses of LPS together with nigericin to induce pyroptosis of THP1 *in vitro*, we found that LPS/nigericin treatment increased the production of proteins and cytokines associated with pyroptosis, including TLR4, NLRP3, ASC1, cleaved caspase-1, IL-1β and GSDMD, whereas downregulated the expression of *circ_0075723* in a manner dependent on dose (**Figures 3C, D**). To further examine the direct impacts of *circ_0075723* in macrophage pyroptosis, we transfected the *circ_007572*3-overexpressing vector (OE-*circ_0075723*) or *circ_0075723* silence vector (sh-*circ_0075723*) to overexpress or knockdown *circ_0075723* expression in LPS/nigericin-treated THP-1 cells (**Figure 3E**). Subsequently, we choose sh2-*circ_0075723* for further experiments due to the relatively lower expression of *circ_0075723* in transfected THP-1 cells than sh1-*circ_0075723* and sh3-*circ_0075723* (**Supplementary Figure 3B**). Overexpression of *circ_0075723* in THP-1 cells showed a strong inhibition of pyroptosis-related proteins and cytokines expression, while silencing of circ_0075723 exhibited the opposite effect (**Figures 3F, G**, **Supplementary Figure 3C**). In general, these studies indicate that macrophages pyroptosis is activated in pneumonia-induced sepsis patients and *circ_0075723* essentially prohibits macrophages pyroptosis *in vitro*.

### *Circ_0075723* functions as a sponge for miR-155-5p in THP-1

**Figure 4:**
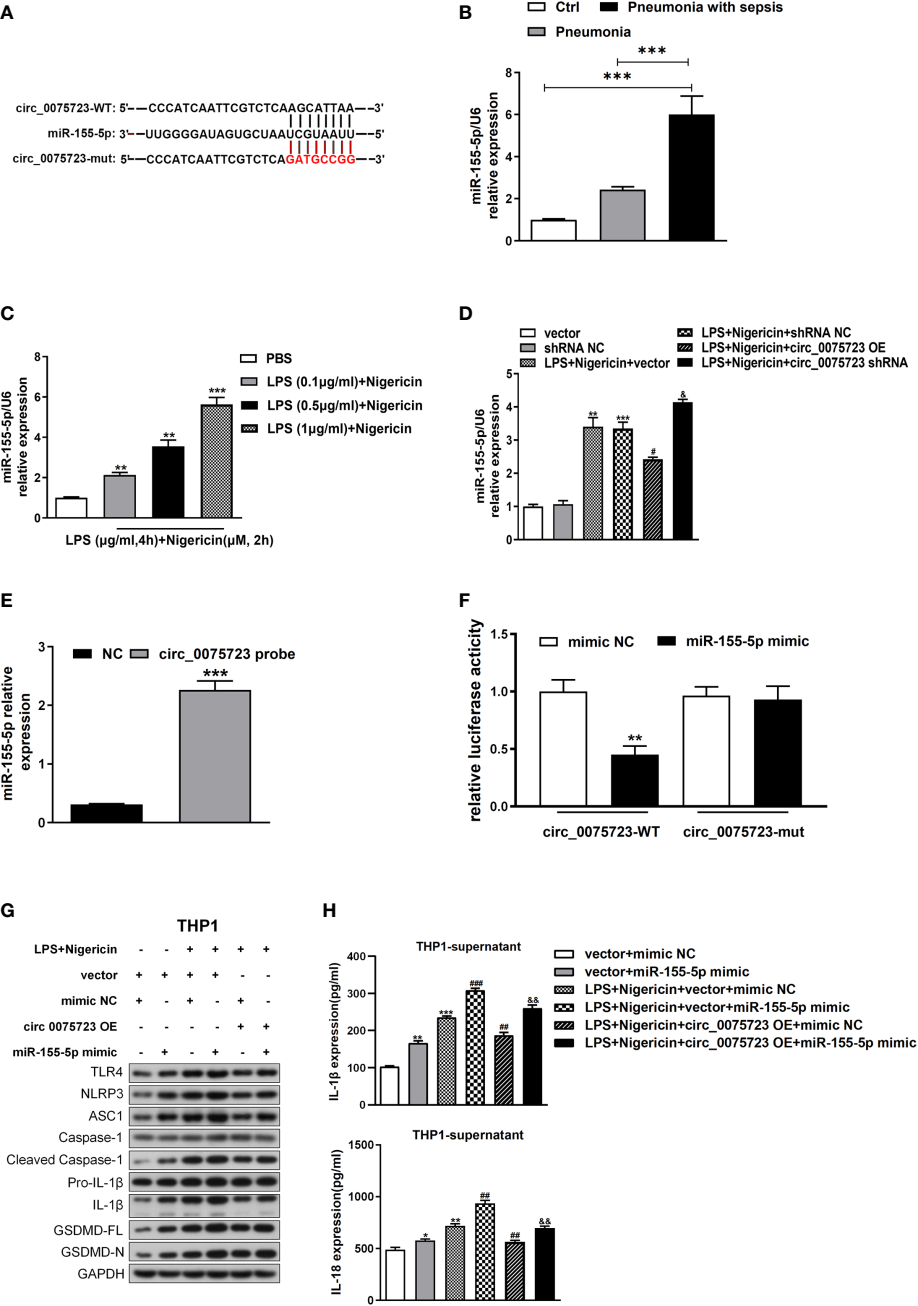
Circ_0075723 functions as a sponge for miR-155-5p in THP-1. **(A)** Schematic showing the predicted miR-155-5p sites in circ_0075723. **(B)** qRT-PCR analysis of the expression of miR-155-5p in CD14+ monocytes among the pneumonia-induced sepsis, pneumonia without sepsis and healthy control group. Each group has 4 samples. Data are presented as means ± SD; significant difference was identified with one-way ANOVA; ***P < 0.001 vs. Control or Pneumonia. **(C)** qRT-PCR analysis of the expression of miR-155-5p in THP-1 cells primed with different doses of LPS (0.1, 0.5 and 1 μg/ml) for 4 h and stimulated with nigericin (10 μM) for 2h. Data are presented as means ± SD; significant difference was identified with Student *t*-tests. **p < 0.01 vs. PBS; ***p < 0.001 vs. PBS. **(D)** THP-1 cells were transfected with vector or shRNA scrambled control (shRNA NC) or were transfected with circ_0075723-overexpressing lentivirus plasmids (OE-circ_0075723), sh-circ_0075723-expressing lentivirus plasmids (sh-circ_0075723), vector or shRNA scrambled control (shRNA NC) and then were primed with LPS (1 μg/ml) for 4 h and stimulated with nigericin (10 μM) for 2h. qRT-PCR analysis of the expression of miR-155-5p in THP-1 cells. Data are presented as means ± SD; significant difference was identified with Student *t*-tests. **p < 0.01 vs. vector; ***p < 0.001 vs. shRNA NC; #p < 0.05 vs. LPS/nigericin + Vector; &p < 0.05 vs. LPS/nigericin + shRNA NC. **(E)** RNA pull-down analysis of the interaction between miR-155-5p and circ_0075723. Data are presented as means ± SD; significant difference was identified with Student *t*-tests. ***p < 0.001 vs. NC group. **(F)** Dual-luciferase reporter assay was performed to validate the association between miR-155-5p and circ_0075723. Data are presented as means ± SD; significant difference was identified with one-way ANOVA. **p < 0.01 vs. mimic NC group. THP-1 cells were transfected with vector + mimic scrambled control (mimic NC) or vector + miR-155-5p mimic or were transfected with vector + mimic NC, vector + miR-155-5p mimic, mimic NC + circ_0075723 lentivirus plasmids (circ_0075723 OE), or miR-155-5p mimic + circ_0075723 OE and then were primed with LPS (1 μg/ml) for 4 h and stimulated with nigericin (10 μM) for 2h. **(G)** Western blot analysis of TLR4, NLRP3, ASC1, caspase1, cleaved caspase-1, Pro-IL1β, IL-1β, GSDMD and GAPDH in THP-1 cells. **(H)** ELISA of IL-18 and IL-1β in THP-1 supernatant. Data are presented as means ± SD; significant difference was identified with Student *t*-tests. *p < 0.05 vs. vector + mimic NC; **p < 0.01 vs. vector + mimic NC; ***p < 0.001 vs. vector + mimic NC; ##p < 0.01 vs. LPS/nigericin + vector + mimic NC; ###p < 0.001 vs. LPS/nigericin + vector + mimic NC; &&p < 0.01 vs. LPS/nigericin + circ_0075723-OE + mimic NC.

Based on bioinformatic predictions from the miRanda and TargetScan databases, miR-155-5p was predicted to bind with *circ_0075723*, and *circ_0075723*–miR-155-5p network was depicted in **Figure 4A**. Additionally, we found the miR-155-5p level were considerably higher in CD14+ monocytes of pneumonia-induced sepsis patients than that of pneumonia patients without sepsis and healthy people (**Figure 4B**). Intriguingly, miR-155-5p expression was dramatically increased in LPS/nigericin-treated THP-1 cells in dose-dependent manner (**Figure 4C**). Further, miR-155-5p expression was modulated *via circ_0075723* as miR-155-5p was downregulated by OE-*circ_0075723* and upregulated by *sh-circ_0075723* (**Figure 4D**). According to these results, we hypothesized that *circ_0075723* may modulate macrophages pyroptosis by sponging miR-155-5p. Therefore, we performed RNA pull-down assay to verify the specific connection between *circ_0075723* with miR-155-5p and found that miR-155-5p could directly interact with *circ_0075723* (**Figure 4E**). Besides, dual-luciferase reporter assay revealed that miR-155-5p mimics dramatically decreased the activity of the *circ_0075723* wild-type luciferase reporter but had no effect on the Mut luciferase reporter (**Figure 4F**). As we had observed that *circ_0075723* could inhibit macrophages pyroptosis, we further adopted the rescue trials to find that miR-155-5p mimics could partly reduce protective effect of *circ_0075723* on macrophages pyroptosis (**Figures 4G, H**, **Supplementary Figure 4**). Collectively, our findings indicated that *circ_0075723* acts as a “molecular sponge” for miR-155-5p.

### *Circ_0075723*-miR-155-5p ceRNA modulates macrophage pyroptosis by directly regulating SHIP1

**Figure 5:**
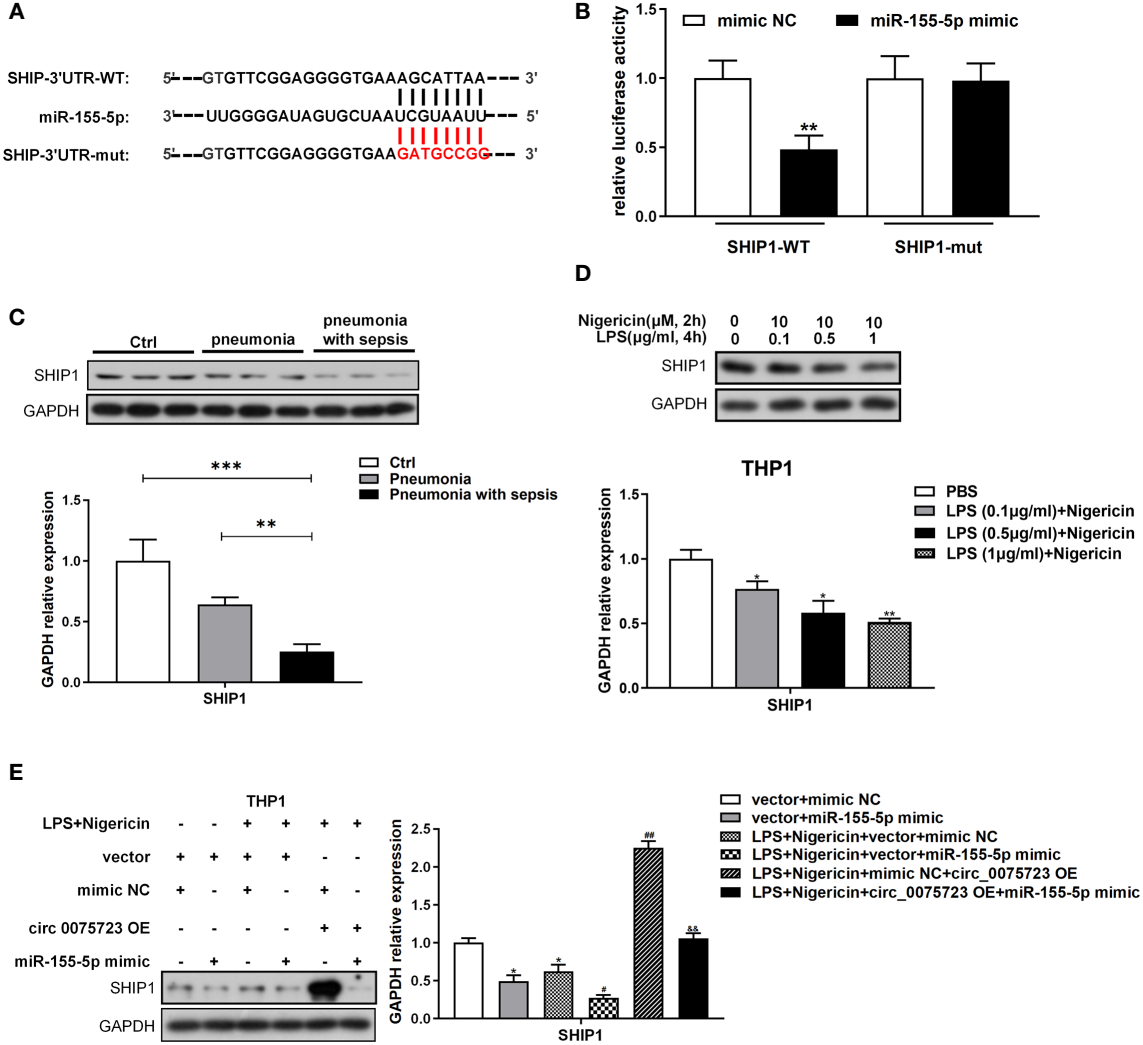
Circ_0075723-miR-155-5p ceRNA modulates macrophage pyroptosis by directly regulating SHIP1. **(A)** Schematic showing the predicted miR-155-5p sites in SHIP1. **(B)** Dual-luciferase reporter assay was performed to validate the association between miR-155-5p and SHIP1. Data are presented as means ± SD; significant difference was identified with one-way ANOVA. **p < 0.01 vs. mimic NC group. **(C)** Western blot analysis of SHIP1 and GAPDH in CD14+ monocytes from pneumonia-induced sepsis, pneumonia without sepsis and healthy control group. Each group has 4 samples. Data are presented as means ± SD; significant difference was identified with one-way ANOVA. **P < 0.01 vs. Pneumonia; ***P < 0.001 vs. Control. **(D)** Western blot analysis of SHIP1 and GAPDH in THP-1 cells primed with different doses of LPS (0.1, 0.5 and 1 μg/ml) for 4 h and stimulated with nigericin (10 μM) for 2h. Data are presented as means ± SD; significant difference was identified with Student *t*-tests. *p < 0.05 vs. PBS; **p < 0.01 vs. PBS. **(E)** THP-1 cells were transfected with vector + mimic scrambled control (mimic NC) or vector + miR-155-5p mimic or were transfected with vector + mimic NC, vector + miR-155-5p mimic, mimic NC + circ_0075723 lentivirus plasmids (circ_0075723 OE) or miR-155-5p mimic + circ_0075723 OE and then were primed with LPS (1 μg/ml) for 4 h and stimulated with nigericin (10 μM) for 2h. Western blot analysis of SHIP1 and GAPDH in THP-1 cells. Data are presented as means ± SD; significant difference was identified with Student *t*-tests. *p < 0.01 vs. vector + mimic NC; #p < 0.05 vs. LPS/nigericin + vector + mimic; ##p < 0.01 vs. LPS/nigericin + vector + mimic; &&p < 0.01 vs. LPS/nigericin + circ_0075723-OE + mimic NC.

To further study the downstream mRNA targets of *circ_0075723*-miR-155-5p ceRNA network, bioinformatic analysis of the TargetScan database revealed that miR-155-5p could target the 3′-untranslated region (UTR) of SHIP1 (**Figure 5A**). By dual-luciferase reporter assay, we showed that miR-155-5p mimics significantly inhibited the wild-type luciferase reporter activity of SHIP1 and validated the connection relationship between SHIP1 and miR-155-5p (**Figure 5B**). Therefore, SHIP1 might be the gene of interest for miR-155-5p. Additionally, SHIP1 expression was markedly diminished in CD14^+^ monocytes of pneumonia-induced sepsis patients compared with that of pneumonia patients without sepsis and healthy people (**Figure 5C**). Corroborating with the clinical findings, vitro tests also confirmed that LPS/nigericin treatment suppressed SHIP1 expression in a dose-dependent way (**Figure 5D**). As it had reported that SHIP1 was involved in the interplay of miR-155 and TLR4 activation by acting as a key negative regulator of TLR4 signaling (23, 25, 26), while TLR4 signaling might activate NLRP3 inflammasome and promote alveolar macrophage pyroptosis (24). We hypothesized that *circ_0075723* might inhibit macrophages pyroptosis through promoting SHIP1 expression by sponging miR-155-5p. To investigate this assertion, we overexpressed miR-155-5p mimics in the LPS/nigericin activated THP-1 cells and found that SHIP1 expression was increased in the company of the *circ_0075723* overexpression, meanwhile miR-155-5p mimics could markedly decrease SHIP1 upregulation induced by the *circ_0075723* overexpression (**Figure 5E**). Besides, we further transfected SHIP1-overexpression lentivirus vector (OE-SHIP1) and (or) miR-155-5p mimics into the LPS/nigericin activated THP-1 cells, and found that the production of pyroptosis-related proteins and cytokines, such as TLR4, NLRP3, ASC1, cleaved caspase-1, GSDMD, IL-1β and IL-18, were decreased in OE-SHIP1 group, meanwhile miR-155-5p mimics could significantly reverse these effects of SHIP1-overexpression (**Supplementary Figures 5A, B**).

### Overexpression of *Circ_0075723* in macrophages downregulates IL-1β and IL-18 expression and protects endothelial cell integrity

**Figure 6:**
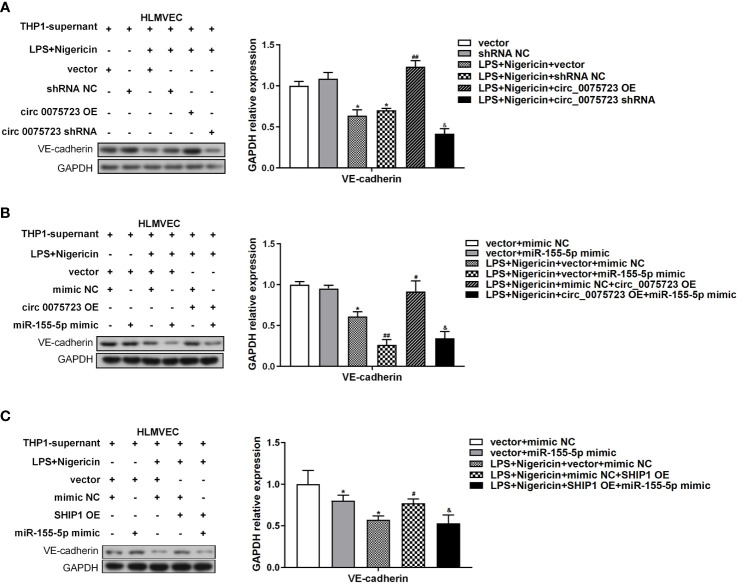
Circ_0075723 in macrophages regulate endothelial permeability through the inhibition expression of IL-1β and IL-18 by circ_0075723/miR-155-5p/SHIP1 axis. **(A)** Western blot analysis of VE-cadherin in HLMVEC cultured with the supernatant for 24 h. The supernatant was collected from aforementioned-LPS-primed THP-1 stimulated with nigericin bearing altered expression of circ_0075723. Data are presented as means ± SD; significant difference was identified with Student *t*-tests. *p < 0.05 vs. Vector or shRNA NC; ##p < 0.01 vs. LPS/nigericin + Vector; &p < 0.05 vs. LPS/nigericin + shRNA NC. **(B)** Western blot analysis of VE-cadherin in HLMVEC cultured with the supernatant for 24 h. The supernatant was collected from aforementioned-LPS-primed THP-1 stimulated with nigericin bearing altered expression of circ_007572 and miR-155-5p. Data are presented as means ± SD; significant difference was identified with Student *t*-tests. *p < 0.05 vs. vector + mimic NC; #p < 0.05 vs. LPS/nigericin + vector + mimic NC; ##p < 0.01 vs. LPS/nigericin + vector + mimic NC; &p < 0.05 vs. LPS/nigericin + mimic NC + circ_0075723-OE. **(C)** Western blot analysis of VE-cadherin in HLMVEC cultured with the supernatant for 24 h. The supernatant was collected from aforementioned-LPS-primed THP-1 stimulated with nigericin bearing altered expression of miR-155-5 and SHIP1. Data are presented as means ± SD; significant difference was identified with Student *t*-tests. *p < 0.05 vs. vector + mimic NC; #p < 0.05 vs. LPS/nigericin + vector + mimic NC; &p < 0.05 vs. LPS/nigericin +mimic NC + SHIP1-OE.

The pathological process of sepsis is complex, and the permeability change caused by vascular endothelial cell damage has a significant role in the pathophysiology of sepsis (27). We have shown that *circ_0075723* in macrophages could inhibit pyroptosis-related pro-inflammatory cytokines IL-1β and IL-18 expression by *circ_0075723*/miR-155-5p/SHIP1 axis, and previous studies had documented that NLRP3 inflammasome associated cytokines IL-1β and IL-18 could increase endothelial cells permeability through inhibiting VE-cadherin expression in endothelial cells (28, 29). We further evaluated whether *circ_0075723* in macrophages could modulate VE-cadherin expression in endothelial cells through inhibiting NLRP3 inflammasome associated cytokines IL-1β and IL-18 by *circ_0075723*/miR-155-5p/SHIP1 axis. To examine the proposition, we collected supernatant from aforementioned-LPS/nigericin activated THP-1 cells bearing altered expression of *circ_0075723*, miR-155-5p and SHIP1, and then used them to culture human lung microvascular endothelial cells (HLMVEC) *in vitro*. Firstly, Western blotting outcomes demonstrated that VE-cadherin expression in HLMVEC cells was downregulated culturing in supernatant harvested from LPS/nigericin-treated THP-1 cells in contrast to control and further dramatically downregulated in supernatant from sh-*circ_0075723*-transfected THP-1 cells, whereas transfected OE-*circ_0075723* THP-1 cells supernatant exhibit the opposite (**Figure 6A**). Besides, the expression trend of VE-cadherin in HLMVEC cells was contrary to the expression of IL-1β and IL-18 in above supernatant (**Figure 3G**). Furthermore, rescue tests showed that the effect of *circ_0075723* and SHIP1 on VE-cadherin expression in HLMVEC cells and IL-1β and IL-18 expression in the supernatant could be inhibited by miR-155-5p mimics (**Figures 4H**,**6B, C**, **Supplementary Figure 5B**). Collectively, these results may indicate that Overexpression *circ_0075723* downregulates IL-1β and IL-18 expression, promotes VE-cadherin expression in endothelial cells and further protects endothelial cell integrity.

## Discussion

Sepsis, especially caused by pneumonia, affects a great many patients all over the world, with high morbidity, mortality and economic expenses (30). Although comprehension of the pathogenesis has grown, and modern therapeutic technologies, such as the use of proper antibiotics, vigorous resuscitation and organ support have also made great progress, the high mortality rate caused by sepsis remains a significant issue (17, 31). Therefore, it is necessary to clarify the potential mechanism to find effective targets for the treatment of pneumonia-induced sepsis. In this investigation, RNA-seq was used to determine the expression profile of circRNAs in the plasma of pneumonia-induced sepsis patients. Through screening the differentially expressed cicRNAs, we identified that *circ_0075723* as a significantly downregulated circRNA in the serum and monocytes of pneumonia-induced sepsis patients as compared to healthy people and pneumonia patients without sepsis. Moreover, we found that *circ_0075723* protected against macrophage pyroptosis through targeting *circ_0075723*-miR-155-5p-SHIP1 axis. In addition, we found that *circ_0075723* suppressed macrophage pyroptosis-induced endothelial permeability by up-regulating VE-cadherin expression. In sum, we firstly determined the influence of *circ_0075723*/miR-155-5p/SHIP1 axis on macrophage pyroptosis, which represents a new mechanism for pneumonia associated sepsis progression. The newly identified *circ_0075723* may be a possible therapeutic target for pneumonia-induced sepsis.

It is generally recognized that sepsis pathophysiology is extremely complex, hence understanding the underlying molecular mechanisms in the occurrence and development of the disease is still a prerequisite to find effective biomarkers and specific treatments to improve survival rate (32). Through regulating the patients’ immune system against different pathogens, circRNAs were revealed to be essential for the pathogenesis of sepsis and sepsis-induced organ dysfunction (33). However, to date, very little clinical research documented specifically expressed circRNAs in the peripheral blood of sepsis patients. Recent research showed the differential expression of circRNAs in lung tissues of patients with sepsis-induced ARDS (34). We also reported the expression of *circN4bp1* in the PBMCs being a diagnostic and predictive marker in ARDS post sepsis (14). In the clinical setting, pneumonia-induced sepsis is one of the most prevalent causes of sepsis and is associated with the greatest fatality rate (3, 4). Therefore, we set out to establish the expression profile of circRNAs using RNA-seq in the plasma of sepsis originated from pneumonia. We discovered that there were variations in plasma circRNA expressions from pneumonia-induced sepsis patients relative to pneumonia patients without sepsis and healthy people, which could contribute to the development and course of the disease. Furthermore, we identified that *circ_0075723* was significantly decreased in the plasma and CD14^+^ monocytes of sepsis patients secondary to pneumonia.

Uncontrolled or excessive inflammation is a hallmark of sepsis. A increasing body of research has shown that pyroptosis, a distinct instance of proinflammatory programmed death, contributes to the excessive inflammatory responses of sepsis and sepsis-related organ damage (35). Therefore, targeting NLRP3 inflammasome activation and the subsequent pyroptosis would be a critical for the therapy of sepsis. Macrophages, as one of the most important cells of the innate immune system, play an important role in inflammatory and immune processes (6). CD14^+^ monocytes are the major subpopulation of monocytes (36), and several clinical studies have shown that changes in the number and function of circulating CD14+ monocytes in patients with sepsis (37–39). However, there have been no reports of CD14+ monocytes pyroptosis in clinical patients with sepsis. Recently, LPS-triggered TLR4 signaling is involved in promoting pulmonary macrophage pyroptosis with activation of NLRP3 inflammasome and elevated expression of the pyroptosis-related proinflammatory cytokines IL-1β and IL-18 (24). Platelet endothelial cell adhesion molecule-1 has been shown to safeguard from sepsis-associated diffuse intravascular coagulation (DIC) through inhibiting macrophage pyroptosis (40). In additional, caspase-11-mediated inflammasome activation and macrophage pyroptosis were controlled by the cAMP metabolism, which attenuated excessive inflammatory responses in sepsis (41). Consistently, we did reveal the upregulation of TLR4, activation of NLRP3 inflammasome, enhanced cleavage of GSDMD, IL-1β and IL-18, and increased release of IL-1β and IL-18 in serum and CD14+ monocytes of pneumonia-induced sepsis patients comparing to pneumonia individuals without sepsis and healthy controls, shedding light on the importance of macrophage pyroptosis in the clinical pathogenesis of sepsis.

Previous studies indicated that circRNAs potentially regulated the macrophage pyroptosis in other clinical conditions, such as ACS (16) and renal fibrosis (15). In our study, we also showed this function in pneumonia-induced sepsis. Since a main way by which circular RNAs exert their effects is by sponging miRNAs *via* ceRNA crosstalk (11), we identified possible miRNAs that interact with *circ_0075723* using bioinformatics. Our study discovered potential regulatory connections between miR-155-5p and SHIP1, as well as between *circ_0075723* and miR-155-5p. The dysregulation of all three genes in pneumonia-induced sepsis patients was confirmed in monocyte/macrophage THP-1 cells. It has been reported that serum exosome-derived miR-155 promoted macrophage proliferation and inflammation involved in sepsis-related acute lung injury (42) and SHIP1 regulated Phagocytosis and M2 Polarization in Pseudomonas aeruginosa Infection as a negative regulator of inflammatory responses (43). Previous studies also documented that SHIP1 is the major target of miR-155 in a wide range of inflammatory diseases (25, 26) and negatively regulates LPS-triggered TLR signaling (22). We discovered that miR-155-5p may bind to the 3′UTR of SHIP1 and suppress its expression, further upregulating the levels of TLR4, thereby activating macrophage pyroptosis as evidenced by the enhanced expression of NLRP3, caspase-1, ASC1, GSDMD and related cytokines as IL-1β and IL-18. In addition, we found that *circ_0075723* could increase SHIP1 expression in macrophage *in vitro*, whereas miR-155-5p mimics could partially counteract this impact. We also confirmed that *circ_0075723* could attenuate the macrophage inflammation and pyroptosis involved in blocking the formation and activation of NLRP3 inflammasome *in vitro*. Therefore, we have revealed a new mechanism of the NLRP3 inflammasome activation through the *circ_0075723*/miR-155-5/SHIP1 axis, which has vital clinical transformation prospects.

One hallmark of acute sepsis is microvessel dysfunction, in which increased endothelial permeability especially lung vascular permeability plays pivotal roles in pneumonia origin sepsis (27, 44). Endothelial permeability is controlled by VE-cadherin, a central component of endothelial adherens junctions (AJs) that modulate the integrity of endothelial junctions and lung fluid balance (45, 46). Previous research demonstrated that pyroptotic immune cells, such as macrophages, release IL-1β and IL-18, which alter vascular integrity and cause organ damage (24, 28). For example, over-released IL-18 caused diabetic retinopathy by increasing retinal vascular permeability (28), and IL-1β could destroy vascular integrity during sepsis-induced lung injury through suppressing VE-cadherin expression in lung endothelial cell (24). Consistent with these findings, we further discovered that *circ_0075723*-miR-155-5p-SHIP1 signaling suppressed IL-1β and IL-18, release from macrophages, which maintained endothelial barrier stability of vascular endothelial cells by repressing the VE-cadherin expression. Future investigations are required to delineate the consequences and the underlying mechanisms of macrophage pyroptosis in mediating endothelial cells permeability of sepsis *in vivo* and further experiments are needed to verify that *circ_0075723* suppressed macrophage pyroptosis in the sepsis mouse model. In summary, our research uncovers a new mechanism by which *circ_0075723* inhibits macrophage pyroptosis and inflammation *via* sponging miR-155-5p, thus enhancing SHIP1 expression. These findings imply that *circ_0075723* may represent a novel therapeutic target for treating pneumonia-induced sepsis.

## Data availability statement

The data presented in the study are deposited in the GEO repository, accession number GSE218494.

## Ethics statement

The studies involving human participants were reviewed and approved by the Research Ethics Board of East Hospital, Tongji University (Shanghai, China). The patients/participants provided their written informed consent to participate in this study.

## Author contributions

DY, DZ, JJ, CW, NL, XB, XL, SJ, and QZ designed, carried out experiments, and performed the genetic analyses. DY, DZ, and JJ wrote the manuscript. LT guided and coordinated the work. All authors contributed to the article and approved the submitted version.
