# Supplementary material for: CircRNA_0075723 protects against pneumonia-induced sepsis through inhibiting macrophage pyroptosis by sponging miR-155-5p and regulating SHIP1 expression
Source: Front Immunol. 2023 Feb 27;14:1095457. doi: 10.3389/fimmu.2023.1095457 (PMC10008927; doi:10.3389/fimmu.2023.1095457)
Supplement: Supplementary file 1 [file Table_1.docx]

|  | TABLE S1 \| participants’ clinical parameters | | | | |
| --- | --- | --- | --- | --- | --- |
| Baseline  Characteristics | | Sepsis  (n=7) | | Pneumonia  (n=7) | Healthy Volunteers  (n=7) |
|  |  |  | Mean ± SD / Median (IQR) | | |
| Age (years) | | 81.21±9.42 | | 71.82±10.31 | 59.62±6.29 |
| Sex [male (%)]  WBC (*10^^9^/L) on day 1  Lymphocyte (*10^^9^/L) on day 1  Neutrophil (*10^9/L) on day 1 | | 4(57.14)  16.18±6.23  0.83±0.31  14.31±6.32 | | 4(57.14)  9.34±2.65  1.2±0.63  8.44±3.82 | 5(71.42)  6.21±3.13  2.49±0.72  4.05±1.07 |
| APACHE II score | | 16.08±7.95 | | 6.28±4.29 | 4.43±1.09 |
| SOFA score on day 1 | | 6.27±3.78 | | 0.61±0.32 | 0 |
| Length of ICU stay(days) | | 9.46(0-41) | | 0 | 0 |
| Length of Hospital stay(days) | | 17.36(2-41.00) | | 11.32(8-17) | 0 |
| Mechanical ventilation (%)  Application of vasoactive drugs (%) | | 4(57.14)  5(71.42) | | 0  0 | 0  0 |
| 28-days mortality (%) | | 2(28.57) | | 0 | 0 |

SD, standard deviation; IQR, inter quartile range; APACHE Ⅱ score, acute physiology and chronic health evaluation Ⅱ score; SOFA, sequential organ failure assessment; P values were calculated by Mann-Whitney U test, Students’-test or one-way analysis of variance (one-way ANOVA), and c²test or Fisher’s exact test, as appropriate. P values below 0.05 indicates statistical signiﬁcance.
