# Supplementary material for: CircRNA_0075723 protects against pneumonia-induced sepsis through inhibiting macrophage pyroptosis by sponging miR-155-5p and regulating SHIP1 expression
Source: Front Immunol. 2023 Feb 27;14:1095457. doi: 10.3389/fimmu.2023.1095457 (PMC10008927; doi:10.3389/fimmu.2023.1095457)
Supplement: Supplementary file 2 [file Table_2.docx]

**Table S2 Primer sequences used for amplification**

| **Name** | **Usage** | **Sequence (5′- 3′)** |
| --- | --- | --- |
| GAPDH  Circ_0075723  U6  NUP153  miR-155-5p | qPCR forward  qPCR reverse  qPCR forward  qPCR reverse  qPCR forward  qPCR reverse  qPCR forward  qPCR reverse  qPCR forward | GGAGCGAGATCCCTCCAAAAT  GGCTGTTGTCATACTTCTCATGG  TGAGGCAAATGTACTACCTCCA  AGGCAGTGAAGAACTGGTGA  CTCGCTTCGGCAGCACA  AACGCTTCACGAATTTGCGT  CAGGGGCCAATTAAGCCTTAC  ACCTCGCTTGTGTCTGTTGAA  TTAATGCTAATCGTGATAGGGGTT |
