# Supplementary material for: CircRNA_0075723 protects against pneumonia-induced sepsis through inhibiting macrophage pyroptosis by sponging miR-155-5p and regulating SHIP1 expression
Source: Front Immunol. 2023 Feb 27;14:1095457. doi: 10.3389/fimmu.2023.1095457 (PMC10008927; doi:10.3389/fimmu.2023.1095457)
Supplement: Supplementary file 3 [file Table_3.docx]

**Table S3 Details of primary antibodies used for immunoblotting analysis**

| **Antibody** | **KD** | **Catalogue number** | **Supplier** | **Dilution** |
| --- | --- | --- | --- | --- |
| SHIP1 | 140 | ab45142 | abcam | 1:1000 |
| TLR4 | 98 | a5258 | abclonal | 1:1000 |
| NLRP3 | 110 | 15101S | cst | 1:1000 |
| ASC1 | 66 | ab70627 | abcam | 1:1000 |
| GSDMD  IL-1β  cleaved-caspase1  caspase1  VE-cadherin | 53, 32  31  20, 22  48  120 | ab210070 ab254360  #4199  #83383  ab33168 | abcam  abcam  cst  cst  abcam | 1:1000  1:1000  1:1000  1:1000  1:1000 |
| GAPDH  Anti-rabbit IgG (HRP) | 37 | #5174  #7074 | cst  cst | 1:20000 1:5000 |
