# Supplementary material for: CircRNA_0075723 protects against pneumonia-induced sepsis through inhibiting macrophage pyroptosis by sponging miR-155-5p and regulating SHIP1 expression
Source: Front Immunol. 2023 Feb 27;14:1095457. doi: 10.3389/fimmu.2023.1095457 (PMC10008927; doi:10.3389/fimmu.2023.1095457)
Supplement: Supplementary file 9 [file DataSheet_1.docx]

**Figure S1**


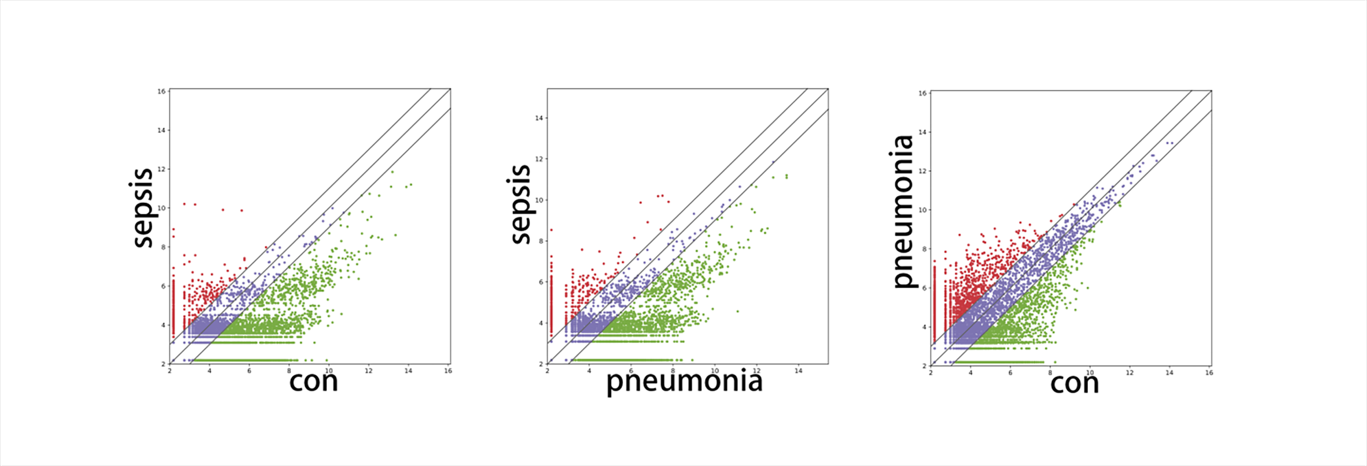


**Figure S1 Specifical expression profiles of circRNAs in pneumonia-induced sepsis**

The scatter plots show the circRNAs expression variation in the plasma from healthy people, pneumonia patients without sepsis and pneumonia-induced sepsis patients. The values of X and Y axes in the scatter plot are the averaged normalized signal values of groups of samples (log2 scaled). The black lines are fold change lines. The circRNAs above the top black line and below the bottom black line indicated more than 2‐fold change of circRNAs between the two compared groups of plasma.
