# Supplementary material for: CircRNA_0075723 protects against pneumonia-induced sepsis through inhibiting macrophage pyroptosis by sponging miR-155-5p and regulating SHIP1 expression
Source: Front Immunol. 2023 Feb 27;14:1095457. doi: 10.3389/fimmu.2023.1095457 (PMC10008927; doi:10.3389/fimmu.2023.1095457)
Supplement: Supplementary file 10 [file DataSheet_2.docx]

**Figure S2**

**
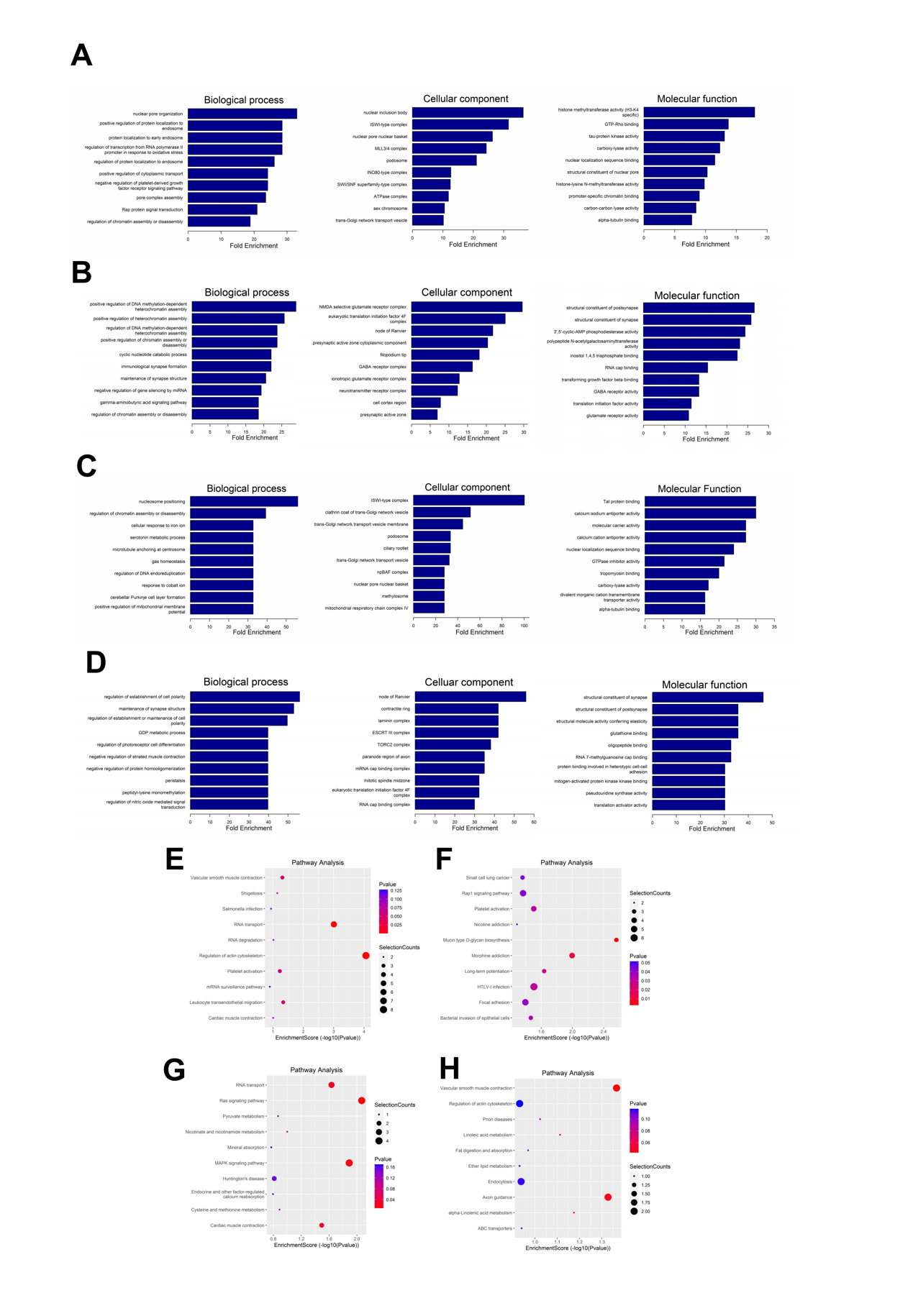
**

**Figure S2 GO and KEGG analysis of the host genes of differentially expressed circRNAs**

GO enrichment analysis for dysregulated circRNAs gene symbols in pneumonia-induced sepsis patients compared with healthy people.

(A) Most significantly enriched GO [–log10 (P value)] terms of downregulated circRNAs gene symbols according to molecular function, cellular component and biological process.

(B) Most significantly enriched GO [–log10 (P value)] terms of upregulated circRNAs gene symbols according to molecular function, cellular component and biological process.

GO enrichment analysis for dysregulated circRNAs gene symbols in pneumonia-induced sepsis patients compared with pneumonia without sepsis patients.

(C) Most significantly enriched GO [–log10 (P value)] terms of downregulated circRNAs gene symbols according to molecular function, cellular component and biological process.

(D) Most significantly enriched GO [–log10 (P value)] terms of upregulated circRNAs gene symbols according to molecular function, cellular component and biological process.

KEGG pathway analysis of the target genes of dysregulated circRNAs. CircRNAs with 2‑fold change and P < 0.05 were selected from the dysregulated circRNAs, and the target genes of these circRNAs were identified using bioinformatics tools.

(E) The top 10 pathways of the target genes of downregulated circRNAs in pneumonia-induced sepsis patients compared with normal healthy people were identified using KEGG analysis according to the enrichment score.

(F) The top 10 pathways of the target genes of upregulated circRNAs in pneumonia-induced sepsis patients compared with normal healthy people were identified using KEGG analysis according to the enrichment score.

(G) The top 10 pathways of the target genes of downregulated circRNAs in pneumonia-induced sepsis patients compared with pneumonia without sepsis patients were identified using KEGG analysis according to the enrichment score.

(H) The top 10 pathways of the target genes of upregulated circRNAs iin pneumonia-induced sepsis patients compared with pneumonia without sepsis patients were identified using KEGG analysis according to the enrichment score.
