# Supplementary material for: CircRNA_0075723 protects against pneumonia-induced sepsis through inhibiting macrophage pyroptosis by sponging miR-155-5p and regulating SHIP1 expression
Source: Front Immunol. 2023 Feb 27;14:1095457. doi: 10.3389/fimmu.2023.1095457 (PMC10008927; doi:10.3389/fimmu.2023.1095457)
Supplement: Supplementary file 11 [file DataSheet_3.docx]

**Figure S3**

**
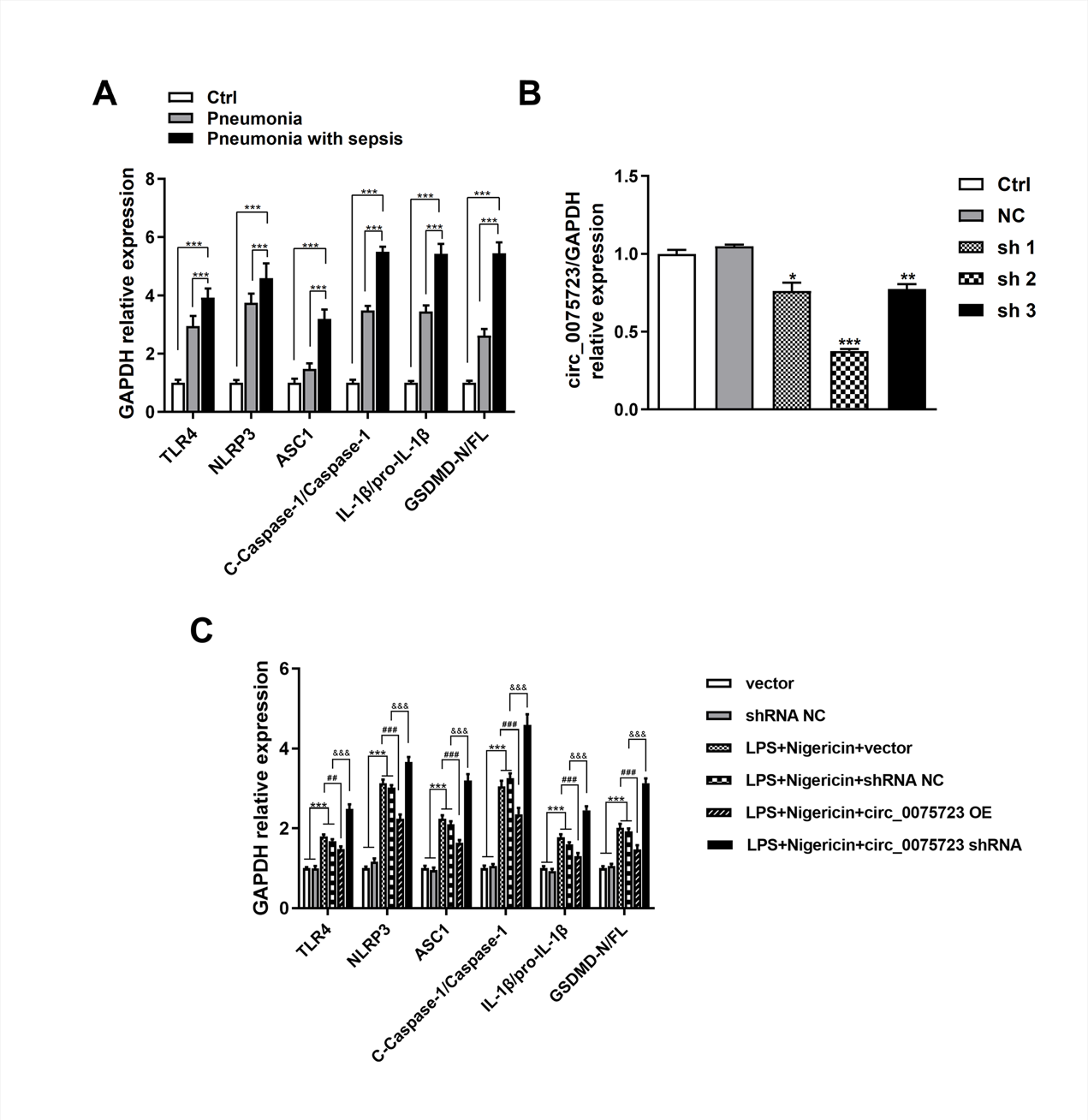
**

**Figure S3 Pyroptosis is activated following pneumonia-induced sepsis and Circ_0075723 inhibits pyroptosis of THP-1 in vitro**

(A) Quantification analysis of expression levels of TLR4, NLRP3, ASC1, caspase1, cleaved caspase-1, Pro-IL1β, IL-1β and GSDMD in Figure 3(A). Data are presented as means ± SD; significant difference was identified with two-way ANOVA. ***p < 0.001 vs. Control or Pneumonia.

(B) The efficiency of sh-circ_0075723 was examined by qRT-PCR, and data are presented as means ± SD; significant difference was identified with Student’s *t*-test. *p < 0.05 vs. Control; **p < 0.01 vs. Control; ***p < 0.001 vs. Control.

(C) Quantification analysis of expression levels of TLR4, NLRP3, ASC1, caspase1, cleaved caspase-1, Pro-IL1β, IL-1β and GSDMD in Figure 3(F). Data are presented as means ± SD; significant difference was identified with two-way ANOVA. ***p < 0.001 vs. vector or shRNA NC; ##p < 0.01 vs. LPS/nigericin + Vector; ###p < 0.001 vs. LPS/nigericin + Vector; &&&p < 0.001 vs. LPS/nigericin + shRNA NC.
