# Supplementary material for: CircRNA_0075723 protects against pneumonia-induced sepsis through inhibiting macrophage pyroptosis by sponging miR-155-5p and regulating SHIP1 expression
Source: Front Immunol. 2023 Feb 27;14:1095457. doi: 10.3389/fimmu.2023.1095457 (PMC10008927; doi:10.3389/fimmu.2023.1095457)
Supplement: Supplementary file 12 [file DataSheet_4.docx]

**Figure S4**

**
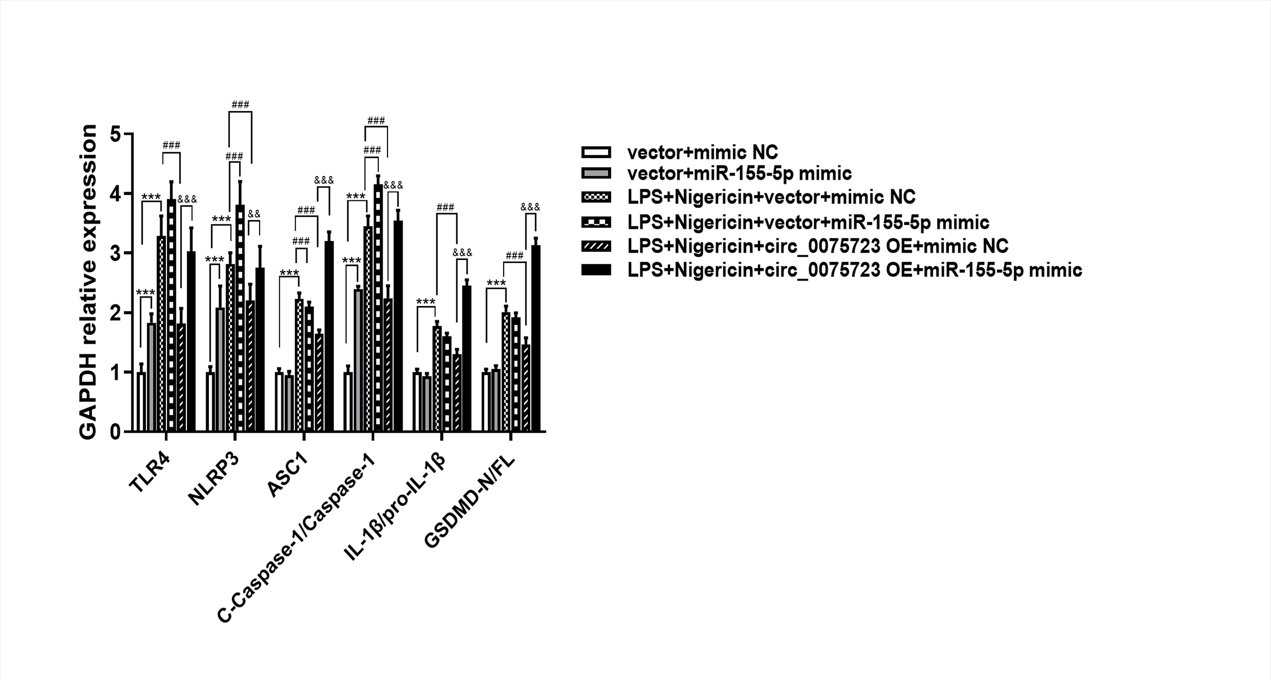
**

**Figure S4 Quantification of expression levels of pyroptosis biomarkers**

Quantification analysis of expression levels of TLR4, NLRP3, ASC1, caspase1, cleaved caspase1, Pro-IL1β, IL-1β and GSDMD. Data are presented as means ± SD; significant difference was identified with two-way ANOVA. ***p < 0.001 vs. vector + mimic NC; ###p < 0.001 vs. LPS/nigericin + vector + mimic NC; &&p < 0.01 vs. LPS/nigericin + circ_0075723-OE + mimic NC; &&&p < 0.001 vs. LPS/nigericin + circ_0075723-OE + mimic NC.
