# Supplementary material for: CircRNA_0075723 protects against pneumonia-induced sepsis through inhibiting macrophage pyroptosis by sponging miR-155-5p and regulating SHIP1 expression
Source: Front Immunol. 2023 Feb 27;14:1095457. doi: 10.3389/fimmu.2023.1095457 (PMC10008927; doi:10.3389/fimmu.2023.1095457)
Supplement: Supplementary file 13 [file DataSheet_5.docx]

**Figure S5**

**
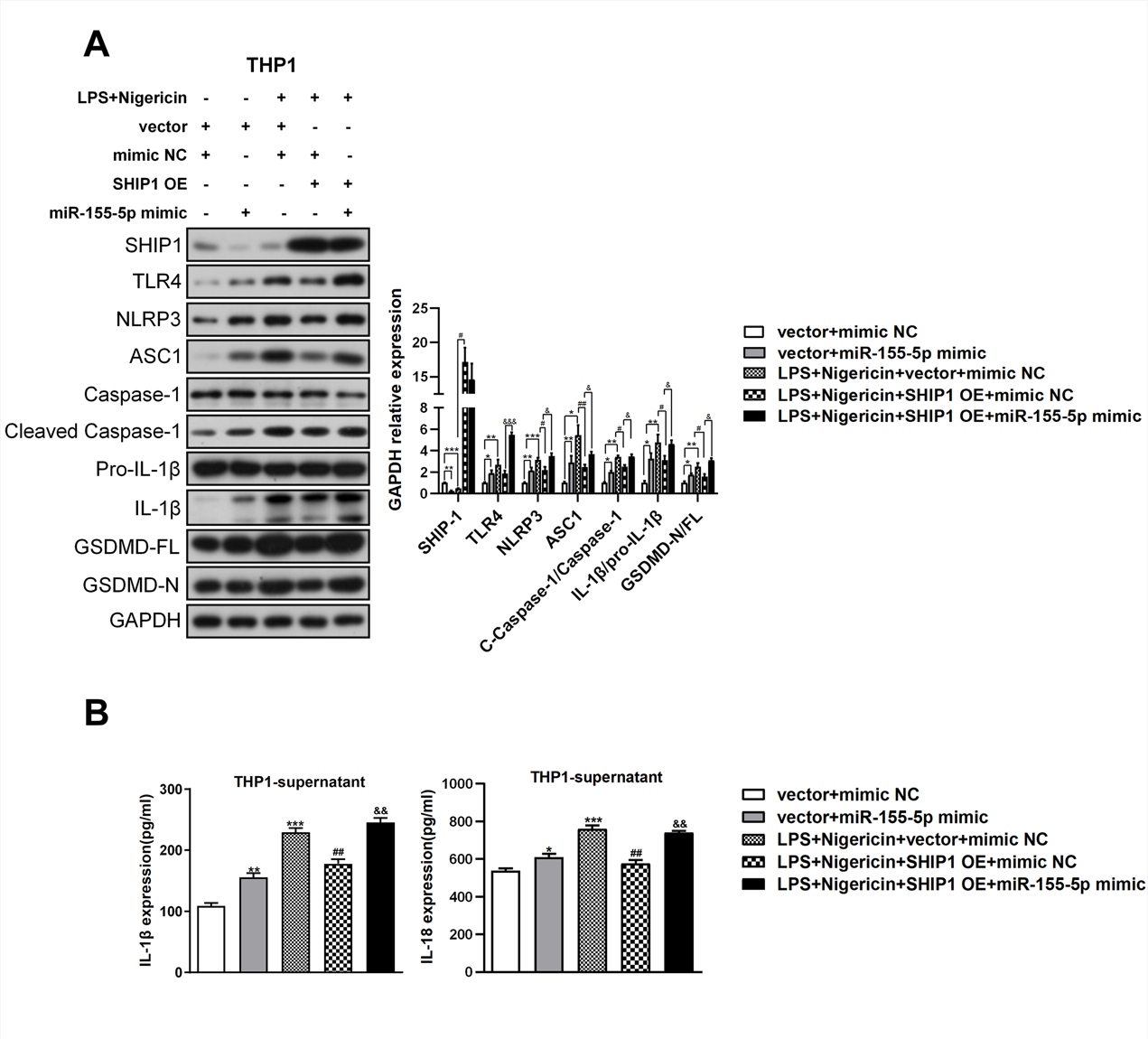
**

**Figure S5 Circ_0075723-miR-155-5p ceRNA modulates macrophage pyroptosis by directly regulating SHIP1**

THP-1 cells were transfected with vector + mimic scrambled control (mimic NC) or vector + miR-155-5p mimic or were transfected with vector + mimic NC, mimic NC + SHIP-OE, or SHIP-OE + miR-155-5p mimic and then were primed with LPS (1 μg/ml) for 4 h and stimulated with nigericin (10 μM) for 2h.

(A) Western blot analysis of SHIP1, TLR4, NLRP3, ASC1, caspase1, cleaved caspase-1, Pro-IL1β, IL-1β, GSDMD and GAPDH in THP-1 cells. Data are presented as means ± SD; significant difference was identified with two-way ANOVA.

(B) ELISA of IL-18 and IL-1β in THP-1 supernatant. Data are presented as means ± SD; significant difference was identified with Student *t*-tests.

*p < 0.05 vs. vector + mimic NC; **p < 0.01 vs. vector + mimic NC; ***p < 0.001 vs. vector + mimic NC; #p < 0.05 vs. LPS/nigericin + vector + mimic NC; ##p < 0.01 vs. LPS/nigericin + vector + mimic NC; &p < 0.05 vs. LPS/nigericin +mimic NC + SHIP1-OE; &&p < 0.01 vs. LPS/nigericin +mimic NC + SHIP1-OE; &&&p < 0.001 vs. LPS/nigericin +mimic NC + SHIP1-OE.
